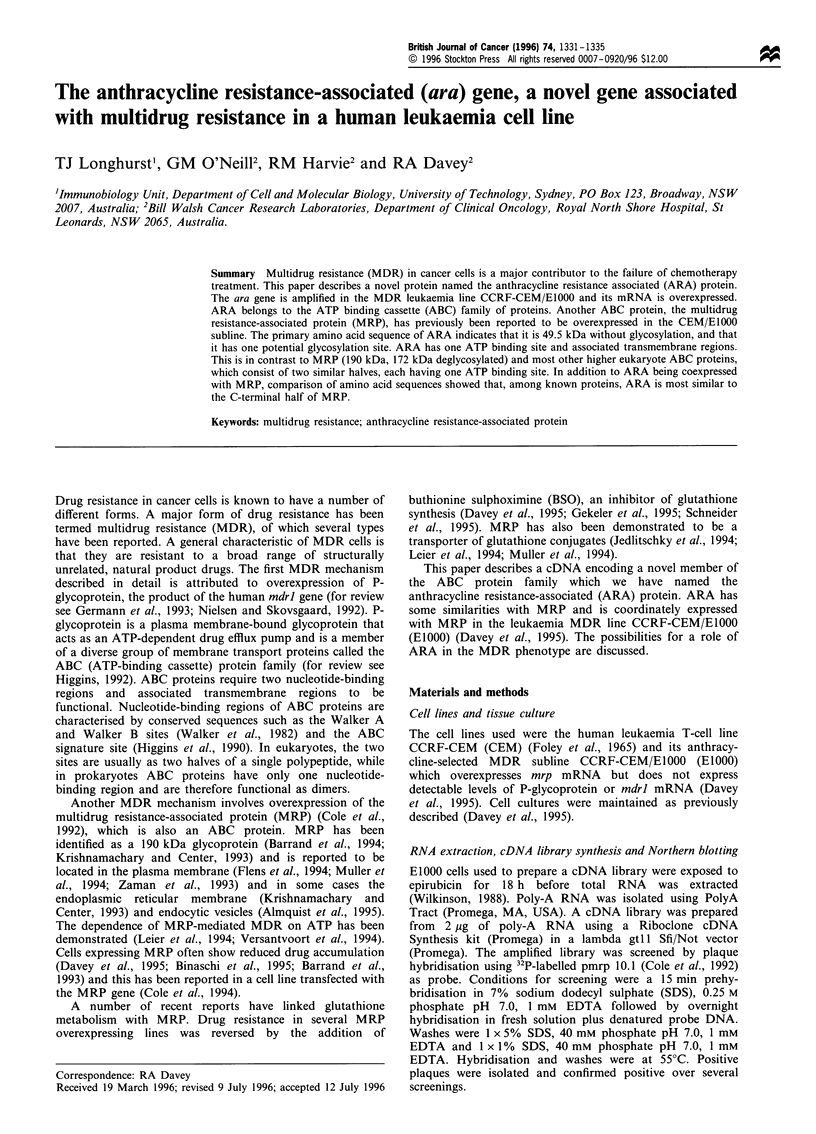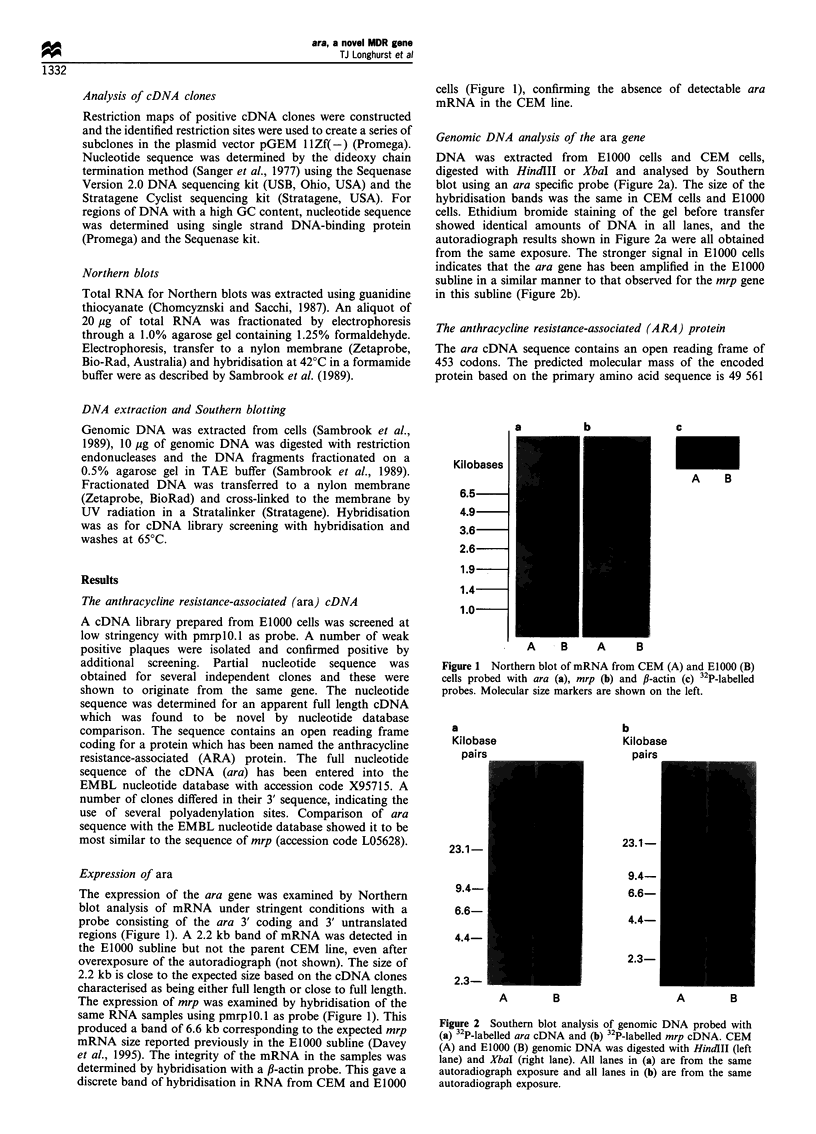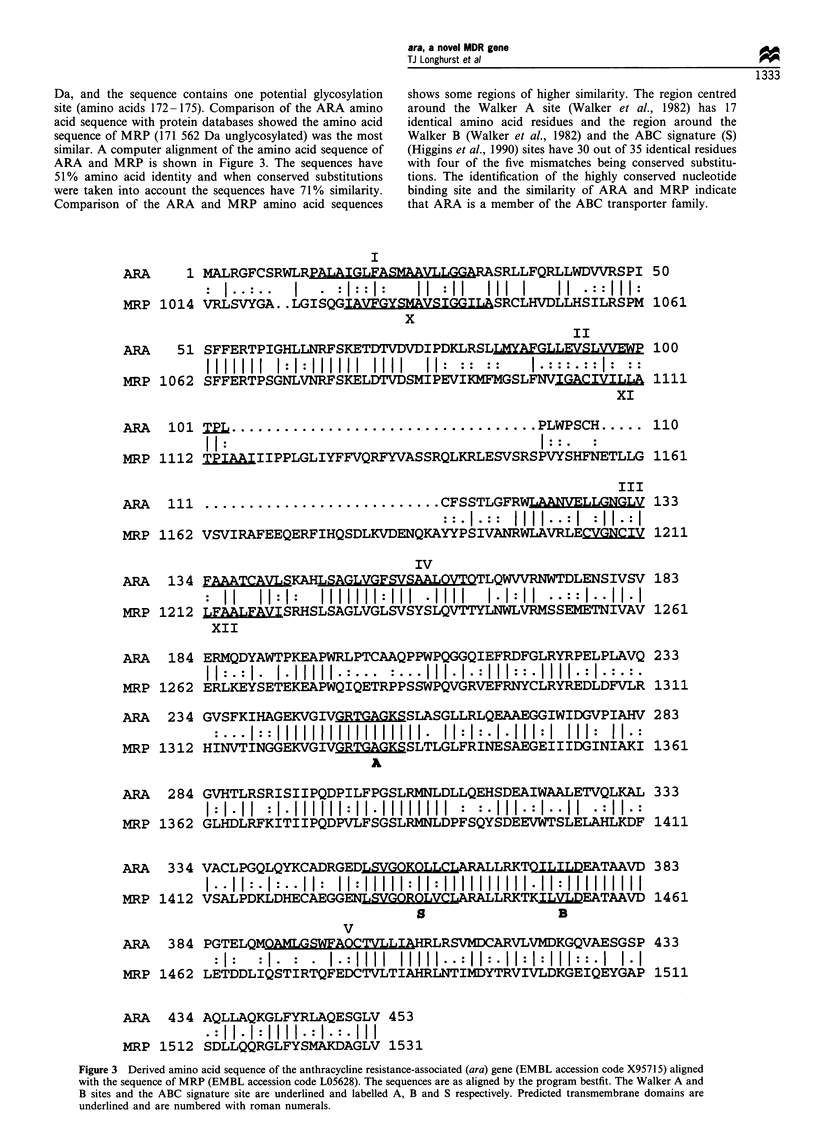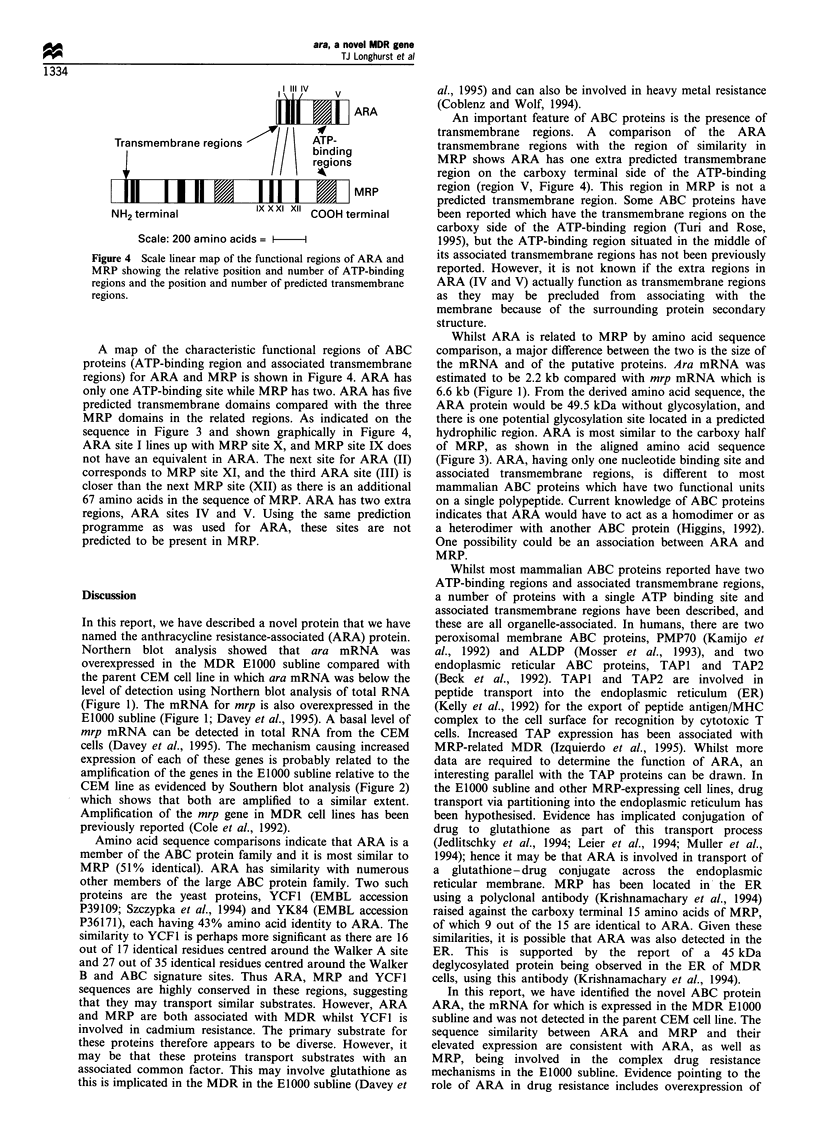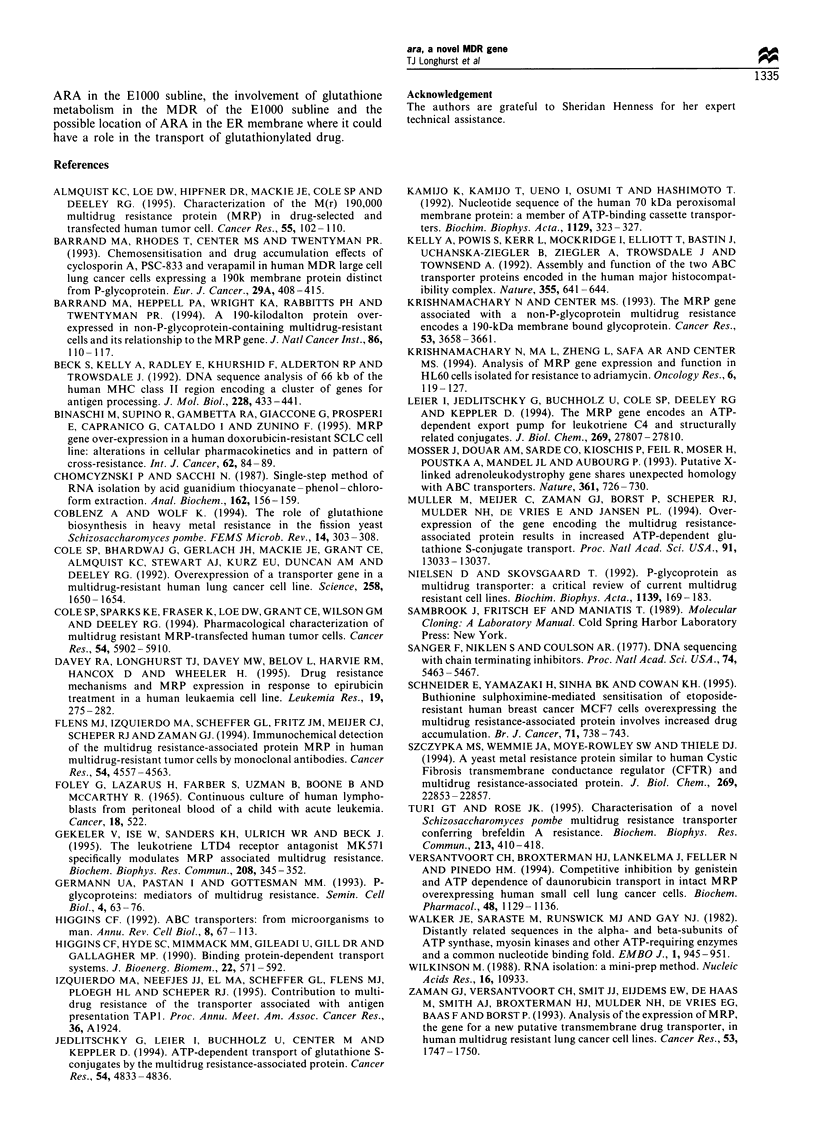# The anthracycline resistance-associated (ara) gene, a novel gene associated with multidrug resistance in a human leukaemia cell line.

**DOI:** 10.1038/bjc.1996.545

**Published:** 1996-11

**Authors:** T. J. Longhurst, G. M. O'Neill, R. M. Harvie, R. A. Davey

**Affiliations:** Department of Cell and Molecular Biology, University of Technology, Sydney, NSW, Australia.

## Abstract

**Images:**


British Journal of Cancer (1996) 74, 1331-1335
© 1996 Stockton Press All rights reserved 0007-0920/96 $12.00

### The anthracycline resistance-associated (*ara*) gene, a novel gene associated with multidrug resistance in a human leukaemia cell line

TJ Longhurst[1], GM O'Neill[2], RM Harvie[2] and RA Davey[2]

[1]*Immunobiology Unit, Department of Cell and Molecular Biology, University of Technology, Sydney, PO Box 123, Broadway, NSW 2007, Australia;* [2]*Bill Walsh Cancer Research Laboratories, Department of Clinical Oncology, Royal North Shore Hospital, St Leonards, NSW 2065, Australia.*

**Summary** Multidrug resistance (MDR) in cancer cells is a major contributor to the failure of chemotherapy treatment. This paper describes a novel protein named the anthracycline resistance associated (ARA) protein. The *ara* gene is amplified in the MDR leukaemia line CCRF-CEM/E1000 and its mRNA is overexpressed. ARA belongs to the ATP binding cassette (ABC) family of proteins. Another ABC protein, the multidrug resistance-associated protein (MRP), has previously been reported to be overexpressed in the CEM/E1000 subline. The primary amino acid sequence of ARA indicates that it is 49.5 kDa without glycosylation, and that it has one potential glycosylation site. ARA has one ATP binding site and associated transmembrane regions. This is in contrast to MRP (190 kDa, 172 kDa deglycosylated) and most other higher eukaryote ABC proteins, which consist of two similar halves, each having one ATP binding site. In addition to ARA being coexpressed with MRP, comparison of amino acid sequences showed that, among known proteins, ARA is most similar to the C-terminal half of MRP.

**Keywords:** multidrug resistance; anthracycline resistance-associated protein

Drug resistance in cancer cells is known to have a number of different forms. A major form of drug resistance has been termed multidrug resistance (MDR), of which several types have been reported. A general characteristic of MDR cells is that they are resistant to a broad range of structurally unrelated, natural product drugs. The first MDR mechanism described in detail is attributed to overexpression of P-glycoprotein, the product of the human *mdr1* gene (for review see Germann *et al.*, 1993; Nielsen and Skovsgaard, 1992). P-glycoprotein is a plasma membrane-bound glycoprotein that acts as an ATP-dependent drug efflux pump and is a member of a diverse group of membrane transport proteins called the ABC (ATP-binding cassette) protein family (for review see Higgins, 1992). ABC proteins require two nucleotide-binding regions and associated transmembrane regions to be functional. Nucleotide-binding regions of ABC proteins are characterised by conserved sequences such as the Walker A and Walker B sites (Walker *et al.*, 1982) and the ABC signature site (Higgins *et al.*, 1990). In eukaryotes, the two sites are usually as two halves of a single polypeptide, while in prokaryotes ABC proteins have only one nucleotide-binding region and are therefore functional as dimers.

Another MDR mechanism involves overexpression of the multidrug resistance-associated protein (MRP) (Cole *et al.*, 1992), which is also an ABC protein. MRP has been identified as a 190 kDa glycoprotein (Barrand *et al.*, 1994; Krishnamachary and Center, 1993) and is reported to be located in the plasma membrane (Flens *et al.*, 1994; Muller *et al.*, 1994; Zaman *et al.*, 1993) and in some cases the endoplasmic reticular membrane (Krishnamachary and Center, 1993) and endocytic vesicles (Almquist *et al.*, 1995). The dependence of MRP-mediated MDR on ATP has been demonstrated (Leier *et al.*, 1994; Versantvoort *et al.*, 1994). Cells expressing MRP often show reduced drug accumulation (Davey *et al.*, 1995; Binaschi *et al.*, 1995; Barrand *et al.*, 1993) and this has been reported in a cell line transfected with the MRP gene (Cole *et al.*, 1994).

A number of recent reports have linked glutathione metabolism with MRP. Drug resistance in several MRP overexpressing lines was reversed by the addition of buthionine sulphoximine (BSO), an inhibitor of glutathione synthesis (Davey *et al.*, 1995; Gekeler *et al.*, 1995; Schneider *et al.*, 1995). MRP has also been demonstrated to be a transporter of glutathione conjugates (Jedlitschky *et al.*, 1994; Leier *et al.*, 1994; Muller *et al.*, 1994).

This paper describes a cDNA encoding a novel member of the ABC protein family which we have named the anthracycline resistance-associated (ARA) protein. ARA has some similarities with MRP and is coordinately expressed with MRP in the leukaemia MDR line CCRF-CEM/E1000 (E1000) (Davey *et al.*, 1995). The possibilities for a role of ARA in the MDR phenotype are discussed.

### Materials and methods

### *Cell lines and tissue culture*

The cell lines used were the human leukaemia T-cell line CCRF-CEM (CEM) (Foley *et al.*, 1965) and its anthracycline-selected MDR subline CCRF-CEM/E1000 (E1000) which overexpresses *mrp* mRNA but does not express detectable levels of P-glycoprotein or *mdr1* mRNA (Davey *et al.*, 1995). Cell cultures were maintained as previously described (Davey *et al.*, 1995).

### *RNA extraction, cDNA library synthesis and Northern blotting*

E1000 cells used to prepare a cDNA library were exposed to epirubicin for 18 h before total RNA was extracted (Wilkinson, 1988). Poly-A RNA was isolated using PolyA Tract (Promega, MA, USA). A cDNA library was prepared from 2 μg of poly-A RNA using a Riboclone cDNA Synthesis kit (Promega) in a lambda gt11 Sfi/Not vector (Promega). The amplified library was screened by plaque hybridisation using $^{32}$P-labelled pmrp 10.1 (Cole *et al.*, 1992) as probe. Conditions for screening were a 15 min prehybridisation in 7% sodium dodecyl sulphate (SDS), 0.25 M phosphate pH 7.0, 1 mM EDTA followed by overnight hybridisation in fresh solution plus denatured probe DNA. Washes were 1 × 5% SDS, 40 mM phosphate pH 7.0, 1 mM EDTA and 1 × 1% SDS, 40 mM phosphate pH 7.0, 1 mM EDTA. Hybridisation and washes were at 55°C. Positive plaques were isolated and confirmed positive over several screenings.

Correspondence: RA Davey
Received 19 March 1996; revised 9 July 1996; accepted 12 July 1996

*Analysis of cDNA clones*

Restriction maps of positive cDNA clones were constructed and the identified restriction sites were used to create a series of subclones in the plasmid vector pGEM 11Zf(−) (Promega). Nucleotide sequence was determined by the dideoxy chain termination method (Sanger *et al.*, 1977) using the Sequenase Version 2.0 DNA sequencing kit (USB, Ohio, USA) and the Stratagene Cyclist sequencing kit (Stratagene, USA). For regions of DNA with a high GC content, nucleotide sequence was determined using single strand DNA-binding protein (Promega) and the Sequenase kit.

*Northern blots*

Total RNA for Northern blots was extracted using guanidine thiocyanate (Chomcyznski and Sacchi, 1987). An aliquot of 20 μg of total RNA was fractionated by electrophoresis through a 1.0% agarose gel containing 1.25% formaldehyde. Electrophoresis, transfer to a nylon membrane (Zetaprobe, Bio-Rad, Australia) and hybridisation at 42°C in a formamide buffer were as described by Sambrook *et al.* (1989).

*DNA extraction and Southern blotting*

Genomic DNA was extracted from cells (Sambrook *et al.*, 1989), 10 μg of genomic DNA was digested with restriction endonucleases and the DNA fragments fractionated on a 0.5% agarose gel in TAE buffer (Sambrook *et al.*, 1989). Fractionated DNA was transferred to a nylon membrane (Zetaprobe, BioRad) and cross-linked to the membrane by UV radiation in a Stratalinker (Stratagene). Hybridisation was as for cDNA library screening with hybridisation and washes at 65°C.

### Results

*The anthracycline resistance-associated (*ara*) cDNA*

A cDNA library prepared from E1000 cells was screened at low stringency with pmrp10.1 as probe. A number of weak positive plaques were isolated and confirmed positive by additional screening. Partial nucleotide sequence was obtained for several independent clones and these were shown to originate from the same gene. The nucleotide sequence was determined for an apparent full length cDNA which was found to be novel by nucleotide database comparison. The sequence contains an open reading frame coding for a protein which has been named the anthracycline resistance-associated (ARA) protein. The full nucleotide sequence of the cDNA (*ara*) has been entered into the EMBL nucleotide database with accession code X95715. A number of clones differed in their 3′ sequence, indicating the use of several polyadenylation sites. Comparison of *ara* sequence with the EMBL nucleotide database showed it to be most similar to the sequence of *mrp* (accession code L05628).

*Expression of* ara

The expression of the *ara* gene was examined by Northern blot analysis of mRNA under stringent conditions with a probe consisting of the *ara* 3′ coding and 3′ untranslated regions (Figure 1). A 2.2 kb band of mRNA was detected in the E1000 subline but not the parent CEM line, even after overexposure of the autoradiograph (not shown). The size of 2.2 kb is close to the expected size based on the cDNA clones characterised as being either full length or close to full length. The expression of *mrp* was examined by hybridisation of the same RNA samples using pmrp10.1 as probe (Figure 1). This produced a band of 6.6 kb corresponding to the expected *mrp* mRNA size reported previously in the E1000 subline (Davey *et al.*, 1995). The integrity of the mRNA in the samples was determined by hybridisation with a β-actin probe. This gave a discrete band of hybridisation in RNA from CEM and E1000 cells (Figure 1), confirming the absence of detectable *ara* mRNA in the CEM line.

*Genomic DNA analysis of the* ara *gene*

DNA was extracted from E1000 cells and CEM cells, digested with *Hind*III or *Xba*I and analysed by Southern blot using an *ara* specific probe (Figure 2a). The size of the hybridisation bands was the same in CEM cells and E1000 cells. Ethidium bromide staining of the gel before transfer showed identical amounts of DNA in all lanes, and the autoradiograph results shown in Figure 2a were all obtained from the same exposure. The stronger signal in E1000 cells indicates that the *ara* gene has been amplified in the E1000 subline in a similar manner to that observed for the *mrp* gene in this subline (Figure 2b).

*The anthracycline resistance-associated (ARA) protein*

The *ara* cDNA sequence contains an open reading frame of 453 codons. The predicted molecular mass of the encoded protein based on the primary amino acid sequence is 49 561

**Figure 1** Northern blot of mRNA from CEM (A) and E1000 (B) cells probed with *ara* (**a**), *mrp* (**b**) and β-actin (**c**) $^{32}$P-labelled probes. Molecular size markers are shown on the left.

**Figure 2** Southern blot analysis of genomic DNA probed with (**a**) $^{32}$P-labelled *ara* cDNA and (**b**) $^{32}$P-labelled *mrp* cDNA. CEM (A) and E1000 (B) genomic DNA was digested with *Hind*III (left lane) and *Xba*I (right lane). All lanes in (**a**) are from the same autoradiograph exposure and all lanes in (**b**) are from the same autoradiograph exposure.

Da, and the sequence contains one potential glycosylation site (amino acids 172–175). Comparison of the ARA amino acid sequence with protein databases showed the amino acid sequence of MRP (171 562 Da unglycosylated) was the most similar. A computer alignment of the amino acid sequence of ARA and MRP is shown in Figure 3. The sequences have 51% amino acid identity and when conserved substitutions were taken into account the sequences have 71% similarity. Comparison of the ARA and MRP amino acid sequences shows some regions of higher similarity. The region centred around the Walker A site (Walker *et al.*, 1982) has 17 identical amino acid residues and the region around the Walker B (Walker *et al.*, 1982) and the ABC signature (S) (Higgins *et al.*, 1990) sites have 30 out of 35 identical residues with four of the five mismatches being conserved substitutions. The identification of the highly conserved nucleotide binding site and the similarity of ARA and MRP indicate that ARA is a member of the ABC transporter family.

**Figure 3** Derived amino acid sequence of the anthracycline resistance-associated (*ara*) gene (EMBL accession code X95715) aligned with the sequence of MRP (EMBL accession code L05628). The sequences are as aligned by the program bestfit. The Walker A and B sites and the ABC signature site are underlined and labelled A, B and S respectively. Predicted transmembrane domains are underlined and are numbered with roman numerals.

**Figure 4** Scale linear map of the functional regions of ARA and MRP showing the relative position and number of ATP-binding regions and the position and number of predicted transmembrane regions.

A map of the characteristic functional regions of ABC proteins (ATP-binding region and associated transmembrane regions) for ARA and MRP is shown in Figure 4. ARA has only one ATP-binding site while MRP has two. ARA has five predicted transmembrane domains compared with the three MRP domains in the related regions. As indicated on the sequence in Figure 3 and shown graphically in Figure 4, ARA site I lines up with MRP site X, and MRP site IX does not have an equivalent in ARA. The next site for ARA (II) corresponds to MRP site XI, and the third ARA site (III) is closer than the next MRP site (XII) as there is an additional 67 amino acids in the sequence of MRP. ARA has two extra regions, ARA sites IV and V. Using the same prediction programme as was used for ARA, these sites are not predicted to be present in MRP.

### Discussion

In this report, we have described a novel protein that we have named the anthracycline resistance-associated (ARA) protein. Northern blot analysis showed that *ara* mRNA was overexpressed in the MDR E1000 subline compared with the parent CEM cell line in which *ara* mRNA was below the level of detection using Northern blot analysis of total RNA (Figure 1). The mRNA for *mrp* is also overexpressed in the E1000 subline (Figure 1; Davey *et al*., 1995). A basal level of *mrp* mRNA can be detected in total RNA from the CEM cells (Davey *et al*., 1995). The mechanism causing increased expression of each of these genes is probably related to the amplification of the genes in the E1000 subline relative to the CEM line as evidenced by Southern blot analysis (Figure 2) which shows that both are amplified to a similar extent. Amplification of the *mrp* gene in MDR cell lines has been previously reported (Cole *et al*., 1992).

Amino acid sequence comparisons indicate that ARA is a member of the ABC protein family and it is most similar to MRP (51% identical). ARA has similarity with numerous other members of the large ABC protein family. Two such proteins are the yeast proteins, YCF1 (EMBL accession P39109; Szczypka *et al*., 1994) and YK84 (EMBL accession P36171), each having 43% amino acid identity to ARA. The similarity to YCF1 is perhaps more significant as there are 16 out of 17 identical residues centred around the Walker A site and 27 out of 35 identical residues centred around the Walker B and ABC signature sites. Thus ARA, MRP and YCF1 sequences are highly conserved in these regions, suggesting that they may transport similar substrates. However, ARA and MRP are both associated with MDR whilst YCF1 is involved in cadmium resistance. The primary substrate for these proteins therefore appears to be diverse. However, it may be that these proteins transport substrates with an associated common factor. This may involve glutathione as this is implicated in the MDR in the E1000 subline (Davey *et al*., 1995) and can also be involved in heavy metal resistance (Coblenz and Wolf, 1994).

An important feature of ABC proteins is the presence of transmembrane regions. A comparison of the ARA transmembrane regions with the region of similarity in MRP shows ARA has one extra predicted transmembrane region on the carboxy terminal side of the ATP-binding region (region V, Figure 4). This region in MRP is not a predicted transmembrane region. Some ABC proteins have been reported which have the transmembrane regions on the carboxy side of the ATP-binding region (Turi and Rose, 1995), but the ATP-binding region situated in the middle of its associated transmembrane regions has not been previously reported. However, it is not known if the extra regions in ARA (IV and V) actually function as transmembrane regions as they may be precluded from associating with the membrane because of the surrounding protein secondary structure.

Whilst ARA is related to MRP by amino acid sequence comparison, a major difference between the two is the size of the mRNA and of the putative proteins. *Ara* mRNA was estimated to be 2.2 kb compared with *mrp* mRNA which is 6.6 kb (Figure 1). From the derived amino acid sequence, the ARA protein would be 49.5 kDa without glycosylation, and there is one potential glycosylation site located in a predicted hydrophilic region. ARA is most similar to the carboxy half of MRP, as shown in the aligned amino acid sequence (Figure 3). ARA, having only one nucleotide binding site and associated transmembrane regions, is different to most mammalian ABC proteins which have two functional units on a single polypeptide. Current knowledge of ABC proteins indicates that ARA would have to act as a homodimer or as a heterodimer with another ABC protein (Higgins, 1992). One possibility could be an association between ARA and MRP.

Whilst most mammalian ABC proteins reported have two ATP-binding regions and associated transmembrane regions, a number of proteins with a single ATP binding site and associated transmembrane regions have been described, and these are all organelle-associated. In humans, there are two peroxisomal membrane ABC proteins, PMP70 (Kamijo *et al*., 1992) and ALDP (Mosser *et al*., 1993), and two endoplasmic reticular ABC proteins, TAP1 and TAP2 (Beck *et al*., 1992). TAP1 and TAP2 are involved in peptide transport into the endoplasmic reticulum (ER) (Kelly *et al*., 1992) for the export of peptide antigen/MHC complex to the cell surface for recognition by cytotoxic T cells. Increased TAP expression has been associated with MRP-related MDR (Izquierdo *et al*., 1995). Whilst more data are required to determine the function of ARA, an interesting parallel with the TAP proteins can be drawn. In the E1000 subline and other MRP-expressing cell lines, drug transport via partitioning into the endoplasmic reticulum has been hypothesised. Evidence has implicated conjugation of drug to glutathione as part of this transport process (Jedlitschky *et al*., 1994; Leier *et al*., 1994; Muller *et al*., 1994); hence it may be that ARA is involved in transport of a glutathione–drug conjugate across the endoplasmic reticular membrane. MRP has been located in the ER using a polyclonal antibody (Krishnamachary *et al*., 1994) raised against the carboxy terminal 15 amino acids of MRP, of which 9 out of the 15 are identical to ARA. Given these similarities, it is possible that ARA was also detected in the ER. This is supported by the report of a 45 kDa deglycosylated protein being observed in the ER of MDR cells, using this antibody (Krishnamachary *et al*., 1994).

In this report, we have identified the novel ABC protein ARA, the mRNA for which is expressed in the MDR E1000 subline and was not detected in the parent CEM cell line. The sequence similarity between ARA and MRP and their elevated expression are consistent with ARA, as well as MRP, being involved in the complex drug resistance mechanisms in the E1000 subline. Evidence pointing to the role of ARA in drug resistance includes overexpression of

ARA in the E1000 subline, the involvement of glutathione metabolism in the MDR of the E1000 subline and the possible location of ARA in the ER membrane where it could have a role in the transport of glutathionylated drug.

**Acknowledgement**
The authors are grateful to Sheridan Henness for her expert technical assistance.

### References

ALMQUIST KC, LOE DW, HIPFNER DR, MACKIE JE, COLE SP AND DEELEY RG. (1995). Characterization of the M(r) 190,000 multidrug resistance protein (MRP) in drug-selected and transfected human tumor cell. *Cancer Res.*, **55**, 102–110.

BARRAND MA, RHODES T, CENTER MS AND TWENTYMAN PR. (1993). Chemosensitisation and drug accumulation effects of cyclosporin A, PSC-833 and verapamil in human MDR large cell lung cancer cells expressing a 190k membrane protein distinct from P-glycoprotein. *Eur. J. Cancer.*, **29A**, 408–415.

BARRAND MA, HEPPELL PA, WRIGHT KA, RABBITTS PH AND TWENTYMAN PR. (1994). A 190-kilodalton protein over-expressed in non-P-glycoprotein-containing multidrug-resistant cells and its relationship to the MRP gene. *J. Natl Cancer Inst.*, **86**, 110–117.

BECK S, KELLY A, RADLEY E, KHURSHID F, ALDERTON RP AND TROWSDALE J. (1992). DNA sequence analysis of 66 kb of the human MHC class II region encoding a cluster of genes for antigen processing. *J. Mol. Biol.*, **228**, 433–441.

BINASCHI M, SUPINO R, GAMBETTA RA, GIACCONE G, PROSPERI E, CAPRANICO G, CATALDO I AND ZUNINO F. (1995). MRP gene over-expression in a human doxorubicin-resistant SCLC cell line: alterations in cellular pharmacokinetics and in pattern of cross-resistance. *Int. J. Cancer*, **62**, 84–89.

CHOMCYZNSKI P AND SACCHI N. (1987). Single-step method of RNA isolation by acid guanidium thiocyanate–phenol–chloroform extraction. *Anal. Biochem.*, **162**, 156–159.

COBLENZ A AND WOLF K. (1994). The role of glutathione biosynthesis in heavy metal resistance in the fission yeast *Schizosaccharomyces pombe*. *FEMS Microb. Rev.*, **14**, 303–308.

COLE SP, BHARDWAJ G, GERLACH JH, MACKIE JE, GRANT CE, ALMQUIST KC, STEWART AJ, KURZ EU, DUNCAN AM AND DEELEY RG. (1992). Overexpression of a transporter gene in a multidrug-resistant human lung cancer cell line. *Science*, **258**, 1650–1654.

COLE SP, SPARKS KE, FRASER K, LOE DW, GRANT CE, WILSON GM AND DEELEY RG. (1994). Pharmacological characterization of multidrug resistant MRP-transfected human tumor cells. *Cancer Res.*, **54**, 5902–5910.

DAVEY RA, LONGHURST TJ, DAVEY MW, BELOV L, HARVIE RM, HANCOX D AND WHEELER H. (1995). Drug resistance mechanisms and MRP expression in response to epirubicin treatment in a human leukaemia cell line. *Leukemia Res.*, **19**, 275–282.

FLENS MJ, IZQUIERDO MA, SCHEFFER GL, FRITZ JM, MEIJER CJ, SCHEPER RJ AND ZAMAN GJ. (1994). Immunochemical detection of the multidrug resistance-associated protein MRP in human multidrug-resistant tumor cells by monoclonal antibodies. *Cancer Res.*, **54**, 4557–4563.

FOLEY G, LAZARUS H, FARBER S, UZMAN B, BOONE B AND MCCARTHY R. (1965). Continuous culture of human lymphoblasts from peritoneal blood of a child with acute leukemia. *Cancer*, **18**, 522.

GEKELER V, ISE W, SANDERS KH, ULRICH WR AND BECK J. (1995). The leukotriene LTD4 receptor antagonist MK571 specifically modulates MRP associated multidrug resistance. *Biochem. Biophys. Res. Commun.*, **208**, 345–352.

GERMANN UA, PASTAN I AND GOTTESMAN MM. (1993). P-glycoproteins: mediators of multidrug resistance. *Semin. Cell Biol.*, **4**, 63–76.

HIGGINS CF. (1992). ABC transporters: from microorganisms to man. *Annu. Rev. Cell Biol.*, **8**, 67–113.

HIGGINS CF, HYDE SC, MIMMACK MM, GILEADI U, GILL DR AND GALLAGHER MP. (1990). Binding protein-dependent transport systems. *J. Bioenerg. Biomem.*, **22**, 571–592.

IZQUIERDO MA, NEEFJES JJ, EL MA, SCHEFFER GL, FLENS MJ, PLOEGH HL AND SCHEPER RJ. (1995). Contribution to multidrug resistance of the transporter associated with antigen presentation TAP1. *Proc. Annu. Meet. Am. Assoc. Cancer Res.*, **36**, A1924.

JEDLITSCHKY G, LEIER I, BUCHHOLZ U, CENTER M AND KEPPLER D. (1994). ATP-dependent transport of glutathione S-conjugates by the multidrug resistance-associated protein. *Cancer Res.*, **54**, 4833–4836.

KAMIJO K, KAMIJO T, UENO I, OSUMI T AND HASHIMOTO T. (1992). Nucleotide sequence of the human 70 kDa peroxisomal membrane protein: a member of ATP-binding cassette transporters. *Biochim. Biophys. Acta.*, **1129**, 323–327.

KELLY A, POWIS S, KERR L, MOCKRIDGE I, ELLIOTT T, BASTIN J, UCHANSKA-ZIEGLER B, ZIEGLER A, TROWSDALE J AND TOWNSEND A. (1992). Assembly and function of the two ABC transporter proteins encoded in the human major histocompatibility complex. *Nature*, **355**, 641–644.

KRISHNAMACHARY N AND CENTER MS. (1993). The MRP gene associated with a non-P-glycoprotein multidrug resistance encodes a 190-kDa membrane bound glycoprotein. *Cancer Res.*, **53**, 3658–3661.

KRISHNAMACHARY N, MA L, ZHENG L, SAFA AR AND CENTER MS. (1994). Analysis of MRP gene expression and function in HL60 cells isolated for resistance to adriamycin. *Oncology Res.*, **6**, 119–127.

LEIER I, JEDLITSCHKY G, BUCHHOLZ U, COLE SP, DEELEY RG AND KEPPLER D. (1994). The MRP gene encodes an ATP-dependent export pump for leukotriene C4 and structurally related conjugates. *J. Biol. Chem.*, **269**, 27807–27810.

MOSSER J, DOUAR AM, SARDE CO, KIOSCHIS P, FEIL R, MOSER H, POUSTKA A, MANDEL JL AND AUBOURG P. (1993). Putative X-linked adrenoleukodystrophy gene shares unexpected homology with ABC transporters. *Nature*, **361**, 726–730.

MULLER M, MEIJER C, ZAMAN GJ, BORST P, SCHEPER RJ, MULDER NH, DE VRIES E AND JANSEN PL. (1994). Overexpression of the gene encoding the multidrug resistance-associated protein results in increased ATP-dependent glutathione S-conjugate transport. *Proc. Natl Acad. Sci. USA.*, **91**, 13033–13037.

NIELSEN D AND SKOVSGAARD T. (1992). P-glycoprotein as multidrug transporter: a critical review of current multidrug resistant cell lines. *Biochim. Biophys. Acta.*, **1139**, 169–183.

SAMBROOK J, FRITSCH EF AND MANIATIS T. (1989). *Molecular Cloning: A Laboratory Manual.* Cold Spring Harbor Laboratory Press: New York.

SANGER F, NIKLEN S AND COULSON AR. (1977). DNA sequencing with chain terminating inhibitors. *Proc. Natl Acad. Sci. USA.*, **74**, 5463–5467.

SCHNEIDER E, YAMAZAKI H, SINHA BK AND COWAN KH. (1995). Buthionine sulphoximine-mediated sensitisation of etoposide-resistant human breast cancer MCF7 cells overexpressing the multidrug resistance-associated protein involves increased drug accumulation. *Br. J. Cancer*, **71**, 738–743.

SZCZYPKA MS, WEMMIE JA, MOYE-ROWLEY SW AND THIELE DJ. (1994). A yeast metal resistance protein similar to human Cystic Fibrosis transmembrane conductance regulator (CFTR) and multidrug resistance-associated protein. *J. Biol. Chem.*, **269**, 22853–22857.

TURI GT AND ROSE JK. (1995). Characterisation of a novel *Schizosaccharomyces pombe* multidrug resistance transporter conferring brefeldin A resistance. *Biochem. Biophys. Res. Commun.*, **213**, 410–418.

VERSANTVOORT CH, BROXTERMAN HJ, LANKELMA J, FELLER N AND PINEDO HM. (1994). Competitive inhibition by genistein and ATP dependence of daunorubicin transport in intact MRP overexpressing human small cell lung cancer cells. *Biochem. Pharmacol.*, **48**, 1129–1136.

WALKER JE, SARASTE M, RUNSWICK MJ AND GAY NJ. (1982). Distantly related sequences in the alpha- and beta-subunits of ATP synthase, myosin kinases and other ATP-requiring enzymes and a common nucleotide binding fold. *EMBO J.*, **1**, 945–951.

WILKINSON M. (1988). RNA isolation: a mini-prep method. *Nucleic Acids Res.*, **16**, 10933.

ZAMAN GJ, VERSANTVOORT CH, SMIT JJ, EIJDEMS EW, DE HAAS M, SMITH AJ, BROXTERMAN HJ, MULDER NH, DE VRIES EG, BAAS F AND BORST P. (1993). Analysis of the expression of MRP, the gene for a new putative transmembrane drug transporter, in human multidrug resistant lung cancer cell lines. *Cancer Res.*, **53**, 1747–1750.